# Novel Immunocytokine IL12-SS1 (Fv) Inhibits Mesothelioma Tumor Growth in Nude Mice

**DOI:** 10.1371/journal.pone.0081919

**Published:** 2013-11-15

**Authors:** Heungnam Kim, Wei Gao, Mitchell Ho

**Affiliations:** Antibody Therapy Section, Laboratory of Molecular Biology, Center for Cancer Research, National Cancer Institute, National Institutes of Health, Bethesda, Maryland, United States of America; University Hospital of Heidelberg, Germany

## Abstract

Mesothelin is a glycosylphosphatidylinositol-anchored glycoprotein that is highly expressed on the cell surface of malignant mesothelioma. Monoclonal antibodies against mesothelin are being evaluated for the treatment of mesothelioma. Immunocytokines represent a novel class of armed antibodies. To provide an alternative approach to current mesothelin-targeted antibody therapies, we have developed a novel immunocytokine based on interleukin-12 (IL12) and the SS1 Fv specific for mesothelin. IL12 possesses potent anti-tumor activity in a wide variety of solid tumors. The newly-developed recombinant immunocytokine, IL12-SS1 (Fv), was produced in insect cells using a baculovirus-insect cell expression system. The SS1 single-chain Fv was fused to the C terminus of the p35 subunit of IL12 through a short linker (GSADGG). The single-chain IL12-SS1 (Fv) immunocytokine bound native mesothelin proteins on malignant mesothelioma (NCI-H226) and ovarian (OVCAR-3) cells as well as recombinant mesothelin on A431/H9 cells. The immunocytokine retained sufficient bioactivity of IL12 and significantly inhibited human malignant mesothelioma (NCI-H226) grown in the peritoneal cavity of nude mice and showed comparable anti-tumor activity to that of the SS1P immunotoxin. IL12-SS1 (Fv) is the first reported immunocytokine to mesothelin-positive tumors and may be an attractive addition to mesothelin-targeted cancer therapies.

## Introduction

Mesothelioma is an asbestos-related cancer that develops from transformed cells originating in the mesothelium. Mesothelin is a differentiation antigen of which expression in normal human tissues is limited to mesothelial cells lining the pleura, pericardium and peritoneum [1,2]. It is highly expressed in several human cancers including mesotheliomas, pancreatic cancers, ovarian cancers, lung adenocarcinomas, intrahepatic cholangiocarcinoma and breast cancer [3-8]. The mesothelin gene (MSLN) encodes a 71-kilodalton (kDa) precursor protein that is processed to a 40-kDa protein termed mesothelin, a glycosyl-phosphatidylinositol-anchored glycoprotein present on the cell surface [1,9]. Mesothelin has been suggested as a promising candidate for targeted therapy of multiple cancers [9].

An immunotoxin, SS1P, that targets mesothelin-expressing tumors has been developed at the US National Cancer Institute (NCI) (Bethesda, MD) and is currently being evaluated in clinical trials [10]. Mice were immunized with a eukaryotic expression vector coding for mesothelin. When high serum antibody titers were obtained, a phage display library was made from the spleen mRNA of these mice. A single-chain variable fragment (scFv)-displaying phage (called SS) was selected that specifically bound to recombinant mesothelin and mesothelin-positive cells [11]. The SS Fv was further improved by *in vitro* affinity maturation [12] and developed as the SS1P immunotoxin [10]. It contains a murine SS1 Fv fused to a 38-kDa fragment of *Pseudomonas* exotoxin A (PE38). Two Phase I clinical studies have been completed at the NCI [13,14]. 

Interleukin-12 (IL12) is a disulfide-linked heterodimeric glycoprotein consisting of a 35-(p35) and a 40-(p40)-kDa subunit [15]. IL12 can enhance the activation of natural killer (NK) cells and cytotoxic T lymphocytes (CTLs), promoting the production of Interferon-gamma (IFN-γ), and inducing the differentiation of T helper cells [16,17]. IL12 also has potent antitumor, anti-angiogenic, and anti-metastatic activities. However, systemic administration of IL12 is thought to be highly toxic, particularly in multiple high doses [18]. In recent years, Dario Neri and colleagues developed IL12-based immunocytokines [19-21]. Delivery of IL12 by a tumor-specific antibody may achieve high and localized IL12 concentrations in the tumor microenvironment, and thereby stimulate and expand the immune effector cells sufficiently to the tumors without causing severe side effects. We think that this strategy could concentrate IL12 in the tumor microenvironment and thereby enhance the antitumor response, presenting an alternative approach for treating patients with mesothelin-overexpressing solid tumors. In this study, we found that IL12-SS1 (Fv) specifically bound mesothelin in several cancer cell lines. We also determined that treatment of mesothelioma tumor-bearing nude mice with IL12-SS1 (Fv) drastically reduced tumor burden in these mice. These results indicate that IL12-SS1 (Fv) may be an effective therapeutic for treating malignant mesothelioma in patients.

## Materials and Methods

### Ethics statement

All mice were housed and treated under the protocol (LMB-059) approved by the Institutional Animal Care and Use Committee at the National Institutes of Health (NIH). 

### Cell lines

The following cell-lines were used in this study: The human mesothelioma cell line NCI-H226, ovarian cancer cell OVCAR-3, and epidermoid carcinoma A431 were obtained from the American Type Culture Collection (ATCC, Rockville, MD). The embryonic kidney cell line, HEK-293, was purchased from Life Technologies (Grand Island, NY). A431/H9: the transfected A431 cell line stably expressing mesothelin was described previously [22]. The cell lines were maintained as adherent monolayer cultures in RPMI 1640 medium (Life Technologies, Gaithersburg, MD) supplemented with 10% fetal bovine serum (FBS) (HyClone, Logan, UT), 1% L-glutamine, and 1% penicillin/streptomycin (Life Technologies, Gaithersburg, MD) and incubated in 5% CO_2_ with a balance of air at 37 °C. Media was changed twice a week to examine the binding properties of IL12-SS1 (Fv). Cells were confirmed to be negative for mycoplasma. The previously generated human mesothelioma cell line LMB-H226-GL was used for the mouse xenograft model [23]. Briefly, we fluorescently labeled the NCI-H226 human mesothelioma cell line by a lentiviral vector harboring a luciferase-GFP (Luc/GFP) fusion gene driven by the RNA polymerase II promoter. After single-cell cloning by flow cytometry, a clone (named LMB-H226-GL) that stably expresses high levels of Luc/GFP was obtained.

### Plasmid construction and cloning of IL12-SS1 (Fv)

Baculovirus cloning, expression, and purification of IL12-SS1 (Fv) was performed by Protein Expression Laboratory, ATP, SAIC-Frederick. pDonr253 is a Gateway Donor vector modified from pDonr201 (Life Technologies, Gaithersburg, MD). pDonr253 replaces the kanamycin resistance gene with a gene encoding spectinomycin resistance. The oligonucleotides used in this study are listed in Table 1.

**Table 1:** Primers used for cloning of IL12-SS1 (Fv).

Primers	Note	Sequences
9228	p40 Forward	5’-ATGTGGGAGCTGGAGAAAGACGTTTATG-3’
9229	p35 Forward	5’-CCGATCCGGTGGCGGTGGCTCGGGCGGTGGTGGGTC GGGTGGCGGCGGATCTAGGGTCATTCCAGTCTCTGGACCTGCC-3’
9230	p40 Reverse	5’-CCGACCCACCACCGCCCGAGCCACCGCCACCGGATC GGACCCTGCAGGGAACACATGCCC-3’
9231	SS1 Forward	5’ – GGCTATCTGAGTTCCGCCGGAAGCGCTGATGGAGGT ATGGCCCAGGTGCAGCTGCAGCAG-3’
9233	SS1 Reverse	5’-GCCCTTGTCGTCATCGTCCTTATAATCGCCCCGTTTTA TTTCCAACTTTGTCCCAGC-3’
9234	p40 Forward	5’-CCATCTCCTGGTTTGCCATCGTTTTGCTGGTGTCTCCA CTCATGGCCATGTGGGAGCTGGAGAAAGACG-3’
9235	p40 Reverse	5’-GGGGACAACTTTGTACAAGAAAGTTGATTAATGGTG ATGGTGATGGTGATGGTGGCCCTTGTCGTCATCGTCC-3’
9237	Adaptor	5’-GGGGACAACTTTGTACAAAAAAGTTGGCACCATGTG TCCTCAGAAGCTAACCATCTCCTGGTTTGCCATCG-3’
9330	p35 Reverse	5’-CGCTTCCGGCGGAACTCAGATAGCCCATCACCCTGTT GATGGTCACGACGCGGGTGCTGAAGGCG-3’

Cloning for IL12-SS1 (Fv). IL12 p40: 9228 + 9230; IL12 p35: 9229 + 9330; scFv SS1: 9231 + 9233 and IL12-SS1 (Fv): 9234 + 9235 + 9237. Primer 9229 and 9230 contained linker (Ser_4_Gly)_3_, 9231 and 9330 contained linker GSADGG, Adaptor 9237 included attB1 sequence.

The subunits of IL12 with SS1 were synthesized (Genescript, Piscataway, NJ) and cloned into pFuse vector (Invivogen, San Diego, CA). We made plasmids pFuse-p40-SS1 and pFuse-SS1-p35 containing p40 and p35 of IL12 with SS1, respectively. The IL12-SS1 (Fv) fragment was constructed using triple overlap PCR from pFuse-p40-SS1 and pFuse-SS1-p35. Three separate PCR reactions were carried out to amplify the IL12 p40, IL12 p35, and SS1 Fv fragments containing 24-28 bp overlapping sequences. Initial PCRs were carried out using Phusion DNA polymerase (New England Biolabs, Ipswich, MA) as noted in Table 1 and per manufacturer’s instructions. The PCR products were purified using the QiaQuick PCR purification kit (Qiagen, Valencia, CA), and equal amounts of the three products were mixed together in a final PCR reaction with the primers noted “IL12-SS1 (Fv)” in Table 1. 

The p40 and p35 subunits of murine IL12 were connected with flexible linker (Ser_4_Gly)_3_. scFv (SS1) was fused to the p35 subunit of IL12 through flexible linker (GSADGG) and IL12-SS1 (Fv), which contains Flag and 8 His tag at the C-terminal. After five cycles of amplification, 200 nM of 9237 adapter primer was added and amplification was continued for 20 additional cycles. The adapter primer, which contains the attB1 site and part of the signal sequence, was used to attach the attB1 site during an adapter PCR process. Conditions were the same as for the original PCR but with an extension time of 1 minute. The final PCR products were flanked by Gateway recombination signal sequences, attB1 at the 5’ end and attB2 at the 3’ end. 

The PCR products were purified using the QiaQuick PCR purification kit (Qiagen, Germantown, MD), and recombined into pDonr253 with the Gateway BP recombination reaction kit following manufacturer’s instruction (Life Technologies, Gaithersburg, MD). *E. coli* DH10B cells were transformed with the BP reactions and colonies were isolated on LB plates containing 50 µg/ml of spectinomycin. The sequence-verified entry clone was subcloned by Gateway LR recombination (Life Technologies, Gaithersburg, MD) into pDest-8 for insect cell expression. Final expression clones were verified by size and restriction digest pattern. *E. coli* DH10Bac (Life Technologies, Gaithersburg, MD) was then transformed with the expression clones and plated on selective media containing gentamycin, kanamycin, tetracycline, IPTG, and X-gal as per the manufacturer’s protocol. White colonies were selected from these plates, and bacmid DNA was recovered by alkaline lysis plasmid preparation and verified by PCR amplification across the bacmid junctions.

### The expression of IL12-SS1 (Fv) in insect cells using baculovirus

The IL12-SS1 (Fv) protein was expressed in baculovirus-infected insect cells. Sf-9 cells were maintained in suspension cultures of Hyclone SFX-Insect medium (Thermo Scientific, Rockford, IL). One day prior to the large-scale direct transfection the Sf-9, cells were fed to ensure that the cells were in logarithmic growth.  On the day of transfection,100 ml of Sf-9 cells per transfection were set at 1.5 x10^6^ cells/ml in a 490 cm^2^ roller bottle (Corning, Tewksbury, MA) and incubated (27°C at 100 rpm) in an INNOVA 4430 shaker (New Brunswick, NJ) while the transfection mixtures were prepared (30 - 60 minutes). Fifty microliters of each bacmid DNA was added to 200 µl of saline in one microcentrifuge tube and 150 µl of transfection reagent (XpressNOW, Lonza, Germany) was added to 200 µl of saline in another tube.  The diluted bacmid DNAs were added to the diluted transfection reagent and the combinations were mixed gently by pipetting. The mixture was incubated for 10 minutes at room temperature and then added to the 100 ml Sf-9 suspension cultures and the bottles were immediately returned to the 27°C shaking incubator. After four days the cultures were centrifuged at 2000 rpm.  The supernatants containing the recombinant baculoviruses were stored at 4°C in the dark. For protein expression, High Five (H5) cells (Life Technologies, Gaithersburg, MD) were fed the day before infection to insure the cells were in logarithmic growth phase the following day. One liter of H5 cells (1.5 x 10^6^ cells/ml) in SFX medium was infected with 40 ml of the p40-p35-SS1 (Fv) virus. The culture was incubated at 21°C for 72 hr at an orbit speed of 100 rpm after which the supernatant was collected for purification.

### Recombinant immunocytokine protein purification

One liter of clarified culture supernatant from baculovirus-infected H5 insect cells was concentrated to 200 ml using a 30 kDa normal molecular weight cutoff (MWCO) membrane (EMD Milipore, Billerica, MA), buffer exchanged to PBS, pH 7.2 (using tangential flow filtration), amended with 50 mM imidazole, and applied to a 5 ml Histrap HP column, (GE Healthcare) that had been equilibrated in PBS, pH 7.2, 50 mM imidazole, at a flow rate of 2 ml/min. The column was washed to baseline with equilibration buffer and proteins eluted in a 20-column volume gradient from 50-500 mM imidazole in PBS, pH 7.2 positive fractions (as identified by SDS-PAGE/Coomassie staining) were pooled and dialyzed to PBS, pH 7.2. Protein concentration was determined by the Bradford assay and bovine IgG as standard (Bio-Rad, Hercules, CA).

### Flow cytometry

To determine binding of IL12-SS1 (Fv) to mesothelin on the cell surface, cancer cells (H226, OVACR-3, H9, A431, and 293-F cells) were grown until confluent, then harvested, washed, and resuspended in ice-cold PBS containing 5 % bovine serum albumin (BSA). Cells were incubated with 5 μg/mL of MN (mesothelin mAb; Rockland Immunochemicals, Gilvertsville, PA) or an isotype antibody and PE-conjugated Anti-Histidin (Abcam, Cambridge, MA). Binding was detected using goat anti-mouse IgG conjugated with phycoerythrin (Sigma-Aldrich, St. Louis, MO). The fluorescence associated with the cells was measured using a FACSCalibur flow cytometer (BD Biosciences, San Jose, CA).

 To evaluate inhibition of the binding of IL12-SS1 (Fv) to A431 cells by an anti-IL12 receptor antibody, 0.5 x 10^6^ A431 cells were incubated with 25 µg/mL of anti-IL12R β2 antibody (Thermo Scientific, Rockford, IL) and incubated on ice for 1 hour. Five µg/milliliter of SS1-IL12 was then added and incubated on ice for an additional hour. After washing, the cells were stained with goat anti-Flag-FITC conjugate (Abcam, Cambridge, MA) at a 1:200 dilution on ice for 1 hour. After washing, the fluorescence associated with the cells was measured by flow cytometry. 

### ELISA

Nunc MaxiSorp 96-well flat-bottomed plates were incubated overnight with 10 to 5000 ng/ml of purified rabbit Fc MSLN (rFc-MSLN) or rabbit Fc control in PBS, followed by an hour block with 5% BSA, 0.01% NaN_3_ in PBS. Purified recombinant IL12-SS1 (Fv) was diluted to 1 μg/ml in ELISA buffer (0.01% Tween 20, 10% SuperBlock, Thermo Scientific, Rockford, IL) and incubated on a plate for 1 hour at room temperature. Plates were then incubated with goat anti-rabbit IgG conjugated HRP for 1 hour at room temperature. The plates were washed four times with ELISA buffer between each coating. Visualization was achieved with 3,3′,5,5′-tetramethylbenzidine detection reagent (KPL, Gaithersburg, MD), and the absorbance was read at 450 nm with a SpectraMax Plus plate reader (Molecular Devices, Sunnyvale, CA). Any absorbance values equal or less than 0.1 are considered negative.

### The bioactivity of IL12-SS1 (Fv)

Peripheral blood mononuclear cells (PBMCs) were isolated from buffy coat of health donors (Department of Transfusion Medicine, NIH Clinical Center) and activated with 10 µg/ml of PH-A for 4 days. Cell proliferation by IL12-SS1 (Fv) or IL12 was measured by a WST-8 colorimetric assay using the Cell Counting Kit-8 (Dojindo Molecular Technologies, Rockville, MD) according to the manufacturer's instructions. Two hundred microliters of PBMCs were seeded at 1 x 10^4^ cells per well in a 96-well plate, and recombinant IL12 or the IL12-SS1 (Fv) fusion proteins were added at the indicated concentrations. Cells were incubated at 37°C for 72 hours, followed by a proliferation assay using Cell Counting Kit-8. The IL12 activity assay was performed with PBMCs in the presence of murine recombinant IL12 or IL12-SS1 (Fv) fusion proteins for 4 days by assessing its ability to induce IFN-γ production using a Human IFN-γ Quantikine ELISA Kit (R&D Systems, Minneapolis, MN). 

### Peritoneal mesothelioma mouse xenograft model

Eight-week old female athymic nude mice (ATHYMIC NCr-nu/nu) obtained from the Animal Production Program at the Frederick National Laboratory for Cancer Research (formerly NCI-Frederick) were housed in micro-isolator cages. To investigate the therapeutic effect of IL12-SS1 (Fv) against established mesothelioma, mesothelioma tumors were established by intraperitoneal (i.p.) injection of 5 million LMB-H226-GL cells in 200 µl of growth media into the low abdomen or flank area of nude mice following our previously reported lab protocol [23]. Animals were imaged the following day and then once every week thereafter. The animal were divided into 4 groups; Vehicle, SS1P (0.4 mg/kg), IL12-SS1 (Fv) (0.4 mg/kg), or IL12-SS1 (Fv) (1.6 mg/kg body weight)-treated mice.

### Animal treatment

We treated the tumor-bearing mice with IL12-SS1 (Fv), SS1P, or vehicle every the other day. The day when the mice were injected with the tumor cells was set as day 1. We used SS1P, an anti-MSLN scFv conjugated with *pseudomonas* toxin, as a positive control. The treatment group and control group each contained 3 mice for initial experiments and 5 mice for repeated experiments. Each mouse in the treatment groups received 0.4 mg/kg body weight of SS1P, 0.4 mg/kg (low dose group), or 1.6 mg/kg (high dose group) of IL12-SS1 (Fv) every other day. The control group received PBS as a vehicle control. Body weight and tumor growth were assessed twice a week. 

### Assessment of tumor growth

Two hundred microliters of 15 mg/mL D-luciferin (Caliper Life Sciences, Hanover, MD) in PBS was injected i.p. before imaging. The luciferase activity of the tumor was calculated using Living Image 3.1.0 software (Caliper Life Sciences, Hanover, MD). Intraperitoneal Tumor growth was assessed using photon intensity, photons per second (ph/sec) as luciferase activity following our lab protocol [23]. 

### *In vivo* toxicology studies

At the end of the treatment, three mice in each group were transferred to SAIC-Frederick (Frederick, MD) and euthanized. Blood was taken for whole blood complete blood counts (CBC) and serum chemistry analysis. A full necropsy was performed, in which organs and tissues were weighed and examined for gross findings. 

### Statistical analysis

Statistical analysis was performed with Prism (version 5) for Windows (GraphPad Software, La Jolla, CA). Raw data were analyzed by “analysis of variance” with Dunnett's and Newman-Keuls multiple comparison post tests. *p* values < 0.05 were considered statistically significant. 

## Results and Discussion

Cytokines are key mediators of innate and adaptive immunity. Even though many cytokines have been used for cancer therapy in patients with advanced cancer, they can sometimes cause severe toxicity [24-27]. The use of ‘immunocytokines’, antibody-cytokine fusion proteins, has been proposed to overcome these problems. In this study, we aim to generate an immunocytokine based on the SS1 anti-mesothelin Fv and investigate whether it is a feasible option for treating mesothelioma and other mesothelin-expressing tumors. 

### Construction of the IL12-SS1 (Fv) immunocytokine

**Figure 1:**
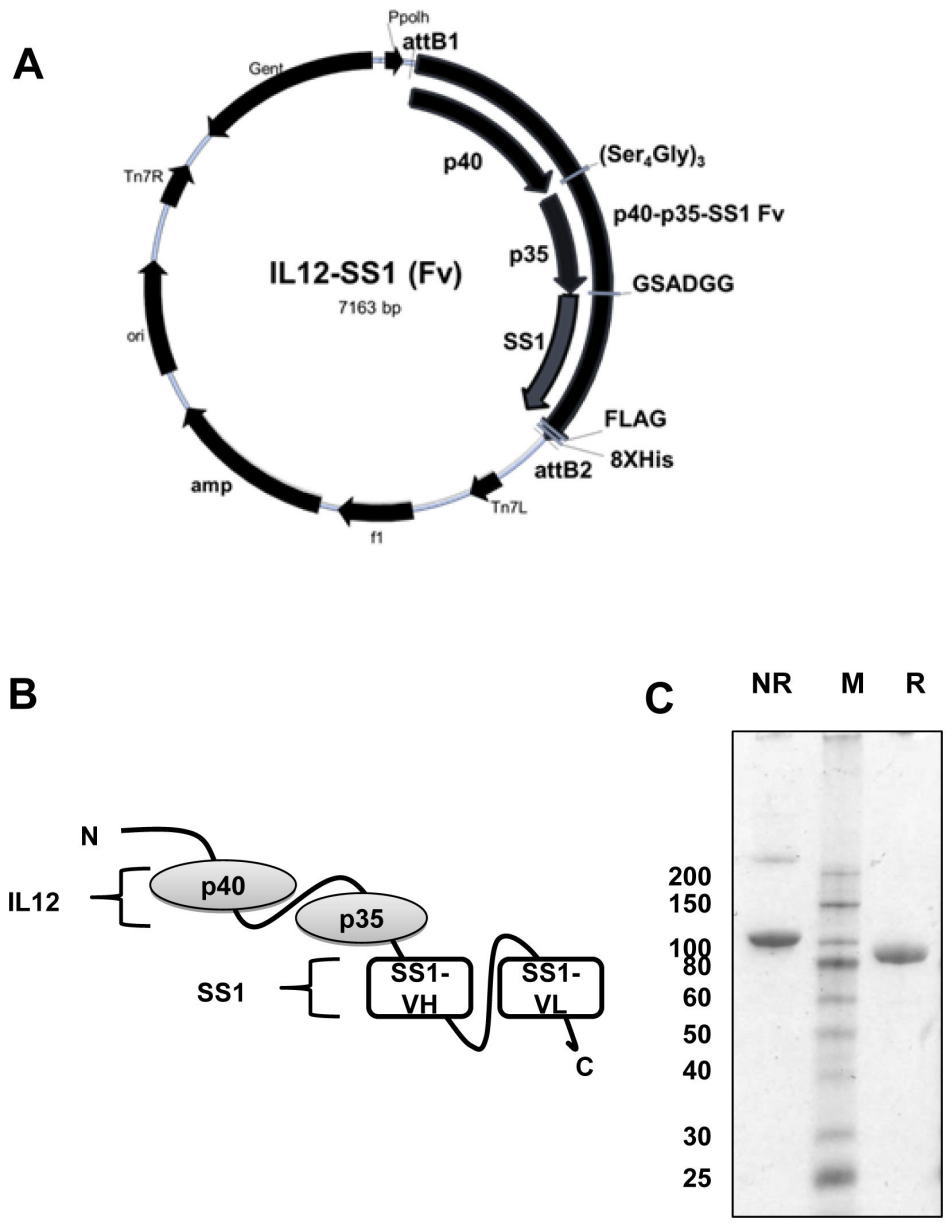
Construction of IL12-SS1 (Fv) fusion protein. (A) The map of the baculovirus expression vector. IL12-SS1 (Fv) was inserted at the recombination sites attB1 and attB2 using the gateway cloning system. (B) Cloning strategy and schematic representation of the IL12-SS1 (Fv) fusion protein. The IL12-SS1 (Fv) fusion protein consists of p40 and p35 fused to scFv (SS1). (C) SDS-PAGE analysis of 5 µg of IL12-SS1 (Fv) protein under reducing (R) or non-reducing (NR) conditions confirms protein size. Analysis: SDS-PAGE, 4-20% gradient gel, Coomassie blue staining. M; protein standards, kDa.

To generate a novel immmunocytokine targeting mesothelin, we constructed monomeric IL12-SS1 (Fv), which is a fusion of IL12 to the anti-mesothelin antibody fragment scFv (SS1). Gafner et al. reported comparative analysis of three fusion proteins (monomeric, heterodimeric and homodimeric protein) comprising scFv (L19) and IL12. Heterodimer protein, which has two subunits consisting of p40 and p35 fused to scFv, showed the most effective tumor uptake and displayed anti-tumor activity superior to monomeric IL12-scFv (L19) in xenograft mouse models [20]. In the present study, we decided to use a similar approach to construct IL12-SS1 (Fv). The p40 and p35 subunits of murine IL12 were connected by flexible linker (Ser_4_Gly)_3_. The scFv (SS1) was fused to the p35 subunit of IL12 through 6 amino acid flexible linker (GSADGG) and IL12-SS1 (Fv) which contains Flag and 8XHis tag at the C-terminal for detection. The final PCR products were flanked by Gateway recombination signal sequences, attB1 at the 5’ end and attB2 at the 3’ end (Figure 1A). Figure 1B depicts the construct containing the p40 and p35 subunits of murine IL12 and the SS1 scFv. IL12-SS1 (Fv) was expressed in sf9 insect cells and fusion protein was purified to homogeneity by affinity chromatography. The production yields were 1.2 mg for IL12-SS1 (Fv) per liter of cell culture supernatant. The resulting purified IL12-SS1 (Fv) protein was characterized by SDS- PAGE analysis, confirming the presence of a single band of apparent molecular weight equal to 90 kDa under reducing and non-reducing conditions (Figure 1C). 

### IL12-SS1 (Fv) specifically binds mesothelin and exerts bioactivity similar to IL12

**Figure 2:**
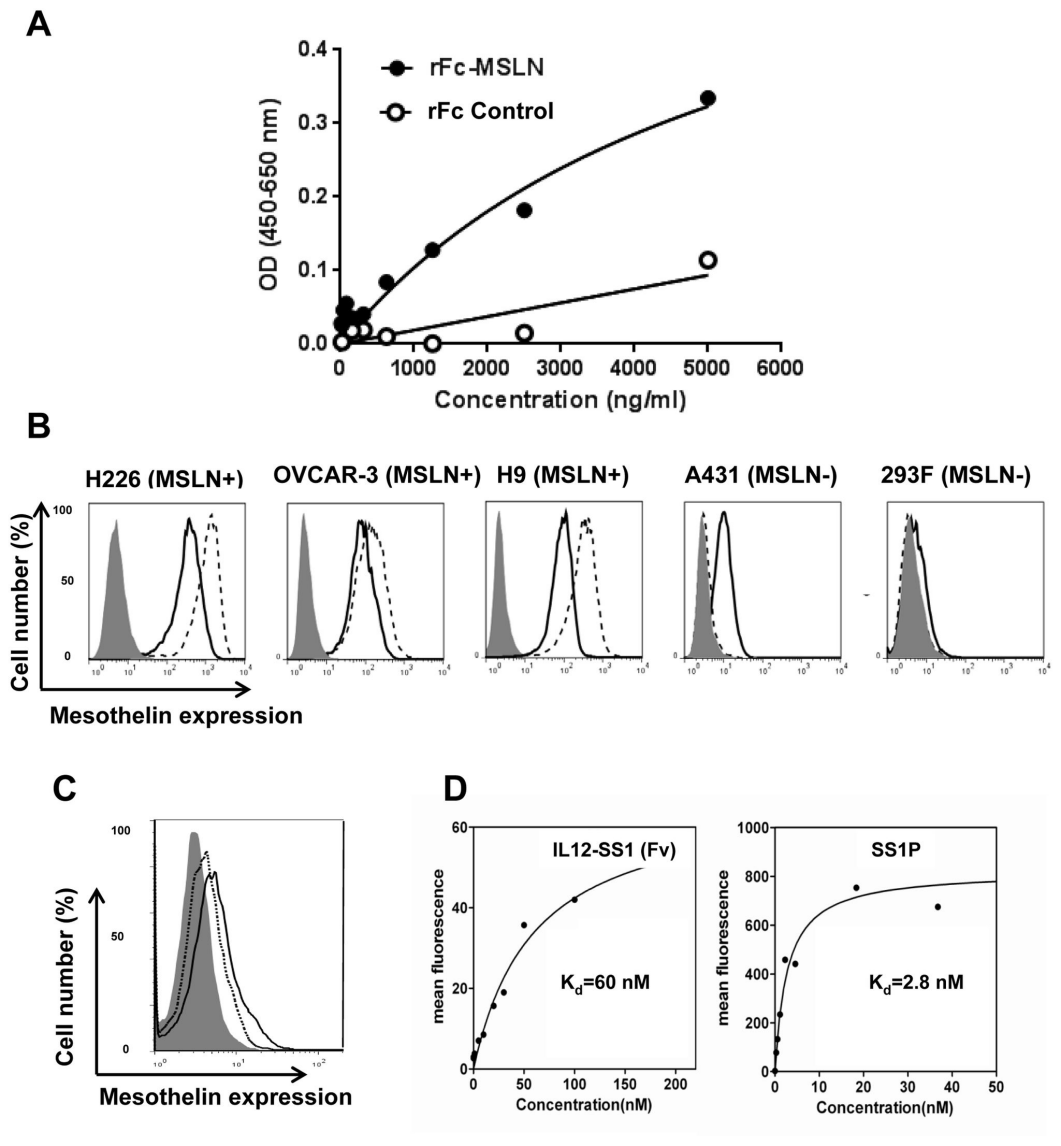
Binding of IL12-SS1 (Fv) to MSLN and cancer cells. (A) The binding specificity of IL12-SS1 (Fv) on MSLN was detected by ELISA. (B) FACS analysis of IL12-SS1 (Fv) on mesothelin-expressing cancer cells. NCI-H226, OVCAR-3, H9, A431, and HEK-293 cells were stained with IL12-SS1 (Fv) which was detected by PE-conjugated anti-histidine antibody (solid line). Mesothelin expression was detected by a mouse anti-MSLN antibody (MN, dotted line) or an irrelevant isotype mAb control (gray surface). Samples were analyzed by flow cytometry. H9: the transfected A431 cell line stably expressing mesothelin; OVCAR-3: human ovarian cancer cell lines; NCI-H226: human mesothelioma cell lines; HEK-293: human embryonic Kidney cell lines. (C) FACS analysis of IL12-SS1 (Fv) on A431 cells. A431 cells were stained with IL12-SS1 (Fv) in the presence of (dotted line) or the absence of (solid line) an anti-IL12R β2 blocking antibody. The binding of IL12-SS1 (Fv) was detected by FITC-conjugated anti-Flag antibody. (D) SS1P and IL12-SS1 (Fv) bound to H9 cells in a dose-dependent manner. H9 cells were stained with indicated concentrations of IL12-SS1 (Fv) and then detected by flow cytometry. The *K*_d_ value for SS1P and IL12-SS1 (Fv) binding to H9 cells are approximately 2.8 and 60 nM, respectively.

To evaluate the binding properties and functionality of the IL12-SS1 (Fv) immunocytokine, we conducted *in vitro* analysis using recombinant proteins and human cancer cells. The binding specificity of IL12-SS1 (Fv) for MSLN was examined by ELISA (Fig. 2A). The recombinant IL12-SS1 (Fv) bound to MSLN in a dose-dependent manner. We used a rabbit Fc (rFc) fusion protein as a negative control and found there was no binding to rFc (OD <=0.1) and no dose-dependent correlation with rFc binding (Fig. 2A). To determine whether the binding of IL12-SS1 (Fv) to the cells was specific, the *in vitro* binding capability of IL12-SS1 (Fv) to MSLN-expressing cells was assessed by flow cytometry (Fig. 2B). Using IL12-SS1 (Fv) for detection, MSLN expression was determined to be strongly positive in NCI-H226, OVCAR-3, and H9 cells, and weakly positive in mesothelin-negative A431 and HEK-293 cells. In the presence of an IL12 receptor blocking antibody, the binding of IL12-SS1 (Fv) to A431 cells was inhibited, indicating that IL12-SS1 (Fv) binds to the IL12 receptor on A431 cells (Figure 2C). In particular, the binding was stronger in H9, an A431 line highly expressing MSLN on the cell surface, than in A431 which is mesothelin-negative. To determine the binding affinity of IL12-SS1 (Fv) for cell surface-associated MSLN, flow cytometric analysis was performed (Figure 2D). We used SS1P as a positive control and found that IL12-SS1 (Fv) and SS1P bound to H9 in a dose-dependent manner. The binding affinity (equilibrium *K*_d_) of IL12-SS1 (Fv) and SS1P for mesothelin-expressing H9 cells were approximately 60 nM and 2.8 nM, respectively. The 20-fold loss of IL12-SS1 (Fv) binding affinity for mesothelin may attribute to the potential interference of single-chain IL12 with SS1. The SS1 single-chain Fv was fused to the C terminus of IL12 through a short linker (GSADGG). It may be worthwhile optimizing the linker sequence in future studies to retain the binding affinity of SS1 (Fv). It is also possible that folding and post-translational modification are different for the two molecules because we used insect cells to express immunocytokine IL12-SS1 (Fv) and *E. coli* for immunotoxin SS1P. 

IL12 is a key immunoregulatory cytokine and plays an essential role in the interactions between the innate and adaptive arms of immunity by acting on natural killer (NK) cells and T cells and enhancing the generation and activity of cytotoxic lymphocytes [28-30]. IL12 is responsible for the priming of Th1 cell responses and the secretion of large amounts of IFN-γ from T cells and NK cells [31]. IL12 induces an antitumor response in a murine model of malignant mesothelioma [32]. IL12 has exhibited potent antitumor and anti-metastatic activity in preclinical studies [31,33-36]. Clinical trials in patients with cancer have revealed promising therapeutic activities, but have also shown that recombinant human IL12 is extremely toxic to humans [37]. Since human IL12 has no biological activity in mice [38], we used mouse IL12 in this study. 

**Figure 3:**
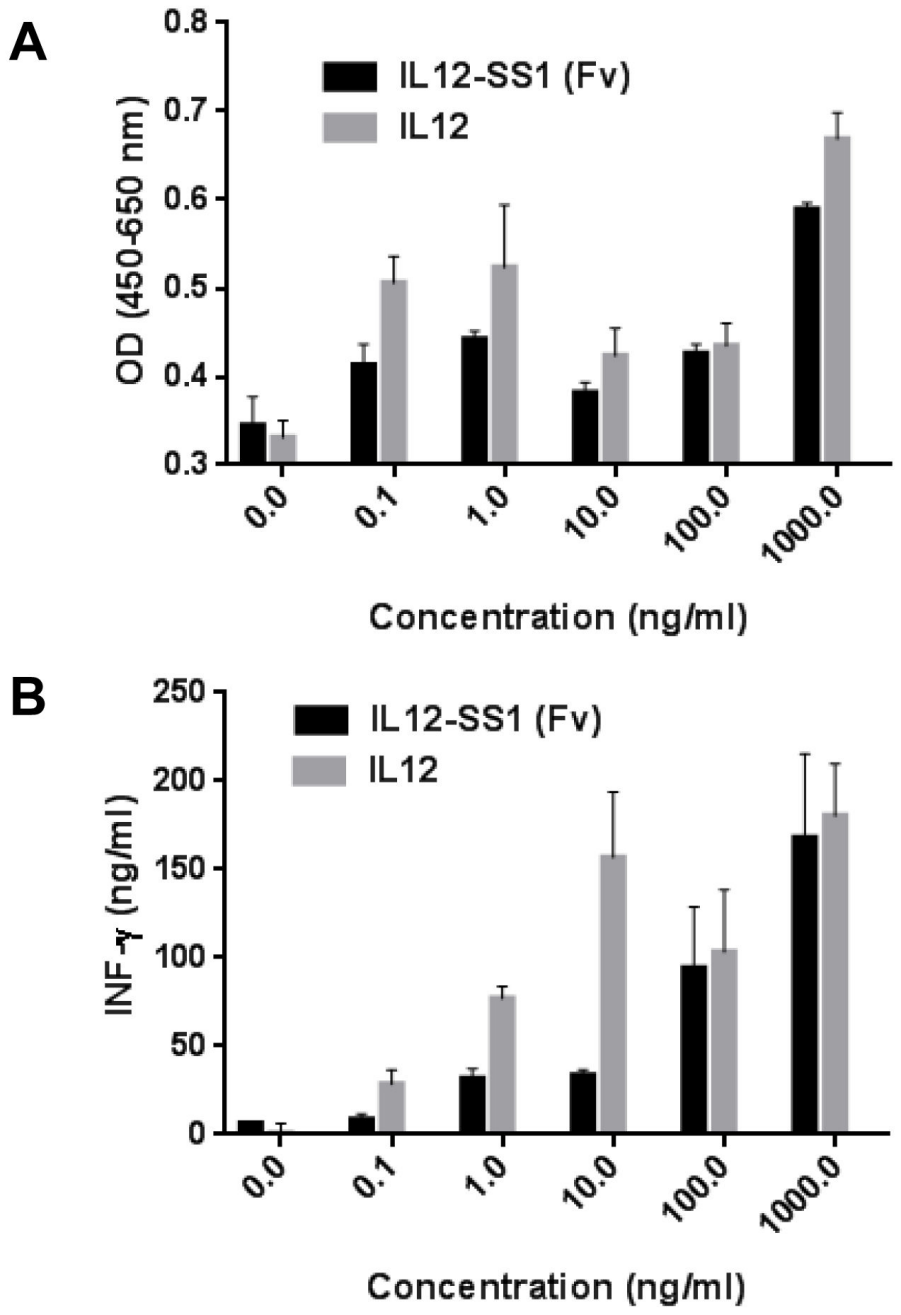
*In vitro* functional analysis. Peripheral blood mononuclear cells (PBMCs) were isolated from buffy coat and activated by 10 µg/ml of PH-A for 4 days. The IL12 activity assay was performed with PBMCs in the presence of murine recombinant IL12 (mIL12) or IL12-SS1 (Fv) fusion proteins for 4 days. (A) Cell proliferation was monitored by a colorimetric assay (WST-8). (B) INF-γ in the supernatant was detected by Human IFN-γ Quantikine ELISA Kit (R&D Systems).

Several studies with tumor specific antibodies fused with IL12 have been reported, including: anti-CD30 antibody for Hodgkin’s lymphoma, anti-HER2 antibody for HER2-expressing tumors [39,40], and anti-extra-domain B (ED-B) of fibronectin for tumor vessels, IL12-huBC1 [41], and IL12-L19 [20]. In order to confirm whether purified IL12-SS1 (Fv) maintains the biological activity of IL12, we compared the stimulatory effect of murine IL12 and IL12-SS1 (Fv) in a lymphocyte proliferation assay, as well as the ability of these proteins to induce INF-γ production in PBMC. Stimulation of PBMCs with doses of IL12 or IL12-SS1 (Fv) as low as 1 ng/ml caused an increase in proliferation (Figure 3A). The specific binding affinity of IL12-SS1 (Fv) to MSLN was 60 nM. Sommavilla et al. have reported about an immunocytokine consisting of the scFv (F8) specific to the extra-domain A of fibronectin and human IL12, F8-IL12, with lymphoproliferative activity at 600 nM [42]. A significant dose-dependent increase in the induction of IFN-γ by treatment with either IL12 or IL12-SS1 (Fv) was noted (Figure 3B). IL12-SS1 (Fv) exhibited comparable biological activities, which was slightly lower than recombinant murine IL12 used as a standard, at high concentrations (100 ng/mL and 1000 ng/mL). At low concentrations (10 ng/mL or less), IL12-SS1 (Fv) exhibited 2- to 4-fold less activity as compared to the control IL12. Taken together, these data indicate that the recombinant IL12-SS1 (Fv) fusion protein exerts its bioactivity by proliferation and induction of IFN-γ production in PBMC.

### IL12-SS1 (Fv) inhibits mesothelioma tumor growth in nude mice

**Figure 4:**
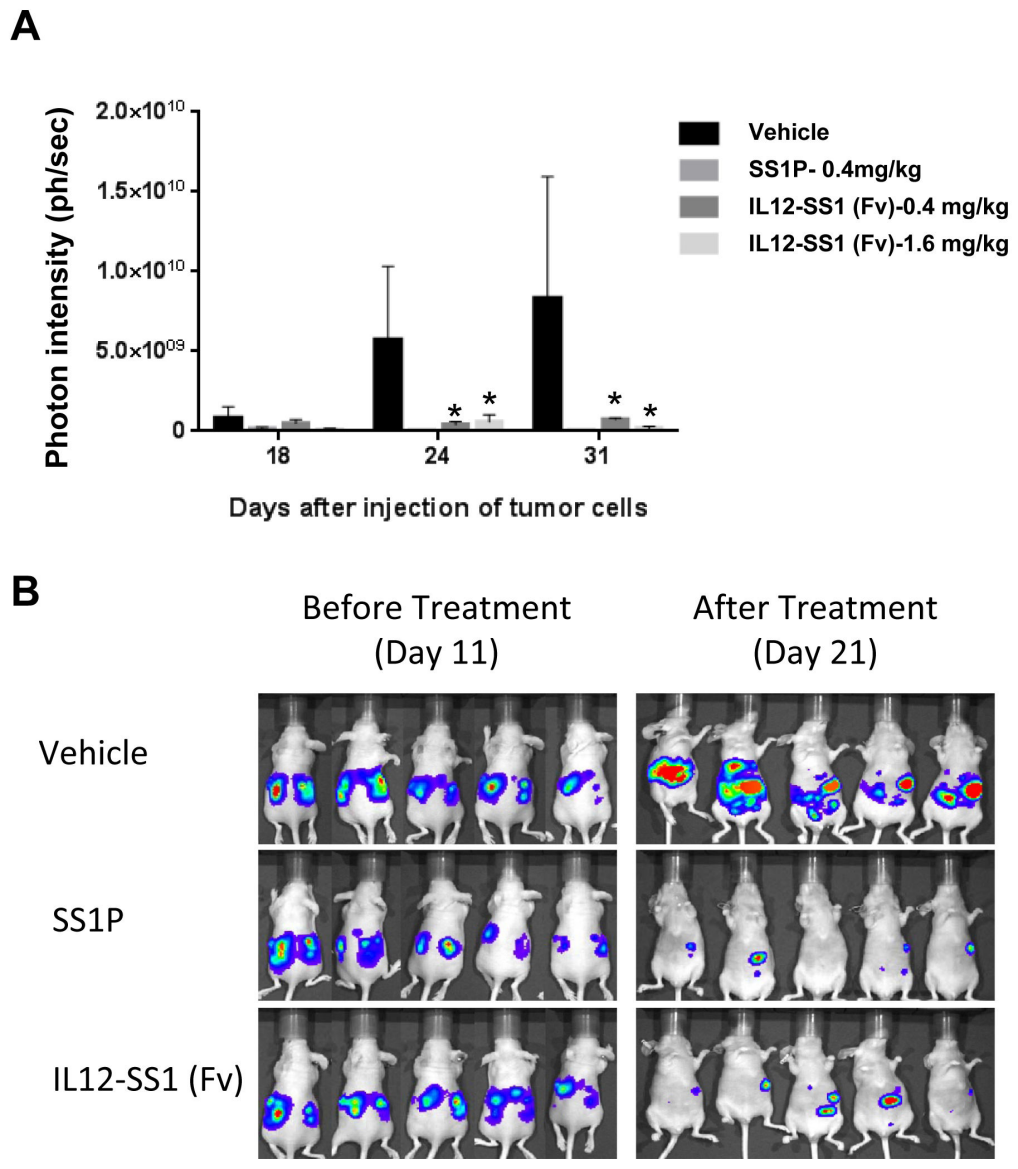
Tumor therapy studies with IL12-SS1 (Fv). (A) Treatment of 8-week old female athymic nude mice with IL12-SS1 (Fv) after intraperitoneal injection of LMB-H226-GL. SS1P (0.4 mg/kg), IL12-SS1 (Fv) (0.4 mg/kg), IL12-SS1 (Fv) (1.6 mg/kg), or vehicle (PBS) were intraperitoneally injected times at days 13, 15, 17, and 19 post-injection. Tumor growth was measured by bioluminescence photometry at day 18, 21, and 31. The photometry of the *in* *vivo* imaging was acquired using Living Image 3.1.0 software (Caliper Life Sciences). *Indicates the significant (*p* < 0.05) difference between treatment and control (vehicle) by using the one-way ANOVA statistical test (GraphPad Prism 5.03). (B) Photograph of representative mice treated with IL12-SS1 (Fv), SS1P and vehicle (PBS).

To investigate the therapeutic effect of IL12-SS1 (Fv) against established mesothelioma, we performed a pilot study in mice. We inoculated mice with LMB-H226-GL (NCI-H226-GFP/Luciferase) mesothelioma cells (Figure 4). In an initial animal experiment, we treated mice with two different doses of IL12-SS1 (Fv), 0.4 mg/kg and 1.6 mg/kg. After 13 days of tumor development, we treated the tumor-bearing mice at days 13, 15, 17, and 19 with 10 or 1.6 mg/kg of IL12-SS1 (Fv), 0.4 mg/kg body weight of SS1P, or a vehicle control. Tumor sizes were accessed via *in vivo* bioluminescence measurement using the IVIS Imaging System. Figure 4A shows that the IL12-SS1 (Fv) and SS1P treatment groups exhibited significantly retarded tumor growth compared with the saline treatment group. In mice treated with vehicle, the proton ranges were gradually increased to 0.8, 5.7 and 8.3 X 10^9^ ph/sec at 18, 24, and 31 days, respectively after i.p. LMB-H226-GL injection. When photons were measured after injections with IL12-SS1 (Fv) or SS1P, the tumor size was significantly reduced. The photons were 0.4, 7.4, and 1.8 X 10^8^ at 31 days after treatment of SS1P (0.4 mg/kg), IL12-SS1 (Fv) (0.4 mg/kg and 1.6 mg/kg), respectively. At Day 18, the tumor sizes in either SS1P or IL12-SS1 (Fv) treated groups were not significantly reduced as compared to the control group (*p* > 0.05). At Day 24 and Day 31, the tumor sizes in the mice treated with either SS1P or IL12-SS1 (Fv) were significantly reduced (*p* < 0.05). Interestingly, the anti-tumor activity of IL12-SS1 (Fv) was similar to that of SS1P (*p* > 0.05). High dose (1.6 mg/kg) or low dose (0.4 mg/kg) of IL12-SS1 (Fv) had similar tumor growth inhibition (*p* > 0.05), indicating that a low dose of the IL12-SS1 (Fv) immunotoxin may also be effective *in vivo*. No significant weight loss was observed in mice treated with vehicle, SS1P, or IL12-SS1 (Fv). In the next animal experiment, we used five mice in each group and 0.4 mg/kg of IL12-SS1 (Fv) (Figure 4B). As shown in the first experiment, both IL12-SS1 (Fv) and SS1P caused tumor remission at Day 21 while the control mice grew large tumors. Collectively, these results suggest that the therapeutic effect of IL12-SS1 (Fv) and its efficacy is comparable to that of the SS1P immunotoxin. 

To examine the toxicity of IL12-SS1 (Fv), we performed *in vivo* toxicology studies at the end of the treatment (Table 2). Blood from mice in treatment and control groups (three mice per group) was analyzed by CBC, and sera isolated for serum chemistry analyses. All serum chemistry and CBC in the IL12-SS1 (Fv)-treated group were similar to those of the control group except white blood cells; the organ weights of IL12-SS1 (Fv) treated-mice were not significantly different from those of the control group mice, indicating that IL12-SS1 (Fv) has no serious toxicity in mice. We noted that the white blood cells were elevated by about 2-old in the IL12-SS1 (Fv) group (16.28 ± 2.47 K/µL) as compared to the control group (7.1 ± 2.16 K/µL) and the SS1P group (7.51 ± 0.97 K/µL). A larger scale study will be needed to investigate the pharmacokinetics, pharmacodynamics and bio-distribution of IL12-SS1 (Fv). 

**Table 2:** Selected *in vivo* toxicological results and organ weights.

**Complete blood counts and serum chemistry Results**					
**Selected parameters**	**Control**	**SS1P**	**IL12-SS1 (Fv)**		**Normal values**
White blood cells (K/µL)	7.1 ± 2.16	7.51 ± 0.97	**16.28 ± 2.47**		1.80 - 10.70
Red blood cells (M/µL)	9.47 ± 0.32	9.51 ± 0.02	10.35 ± 0.84		6.36 - 9.42
Albumin (g/dL)	4.4 ± 0.42	4.27 ± 0.42	4.1 ± 0.3		1.6 - 2.8
Alkaline phosphatase (U/L)	60.5 ± 9.19 13.01	54 ± 15.13	73 ± 4.36		67 - 282
Alanie aminotransferase (U/L)	61 ± 16.52 16.17	46.33 ± 4.51	46.67 ± 12.50		29 - 181
Total bilirubin (mg/dL)	0.3	0.3	0.37 ± 0.06		0.0 - 0.6
Creatinine (mg/dL)	<0.2	<0.2	<0.2		0.2 - 0.4
Hemoglobin (g/dL)	14.53 ± 0.42	14.47 ± 0.65	14.83 ± 0.64		11.00 - 15.10
Total protein (g/dL)	6.67 ± 0.64	6.47 ± 0.15	6.13 ± 0.15		4.2 - 5.9
Blood urea nitrogen (mg/dL)	25.67 ± 3.06	25 ± 2.65	23 ± 1.73		12 - 52
**Organ weights (mg)**					
Brain	0.45 ± 0.04	0.43 ± 0.02	0.43 ± 0.02	NA	
Heart	0.12 ± 0.02	0.12 ± 0.01	0.14 ± 0.01	NA	
Kidney	0.31 ± 0.04	0.29 ± 0.02	0.33 ± 0.02	NA	
Liver	1.02 ± 0.29	1.01 ± 0.05	1.17 ± 0.09	NA	
Lung	0.16 ± 0.03	0.16 ± 0.04	0.16 ± 0.01	NA	
Spleen	0.14 ± 0.12	0.12 ± 0.02	0.12 ±0.06	NA	

NA: not available

In conclusion, we described an IL12-based immunocytokine that targets cell surface-associated mesothelin proteins in mesothelioma. The recombinant immunocytokine produced in baculovirus-insect cell expression system is as biologically active as IL12 alone. Furthermore, our animal testing showed that the immunocytokine exhibited inhibition of human malignant mesothelioma grown in the peritoneal cavity of nude mice. Our results may lead to a new mesothelin-targeted antibody conjugate, which is different from other anti-mesothelin antibodies (such as chimeric antibody MORAb-009) or immunotoxins (SS1P) currently being evaluated in clinical studies. IL12-SS1 (Fv) presents a significant advantage given that local delivery of IL12 to tumor microenvironment by the anti-mesothelin antibody may enhance specific immune response against tumors. Recently, several anti-mesothelin human monoclonal antibodies have been developed [43-45]. Therefore, it is possible to make fully human IL12-based immunocytokines to mesothelin for the treatment of mesothelioma and other mesothelin-expressing tumors in patients. 
